# The interaction effects of risk factors for hypertension in adults: a cross-sectional survey in Guilin, China

**DOI:** 10.1186/s12872-016-0358-4

**Published:** 2016-09-23

**Authors:** Jian Yu, Di-sha Zou, Meng-ting Xie, Yao Ye, Tian-peng Zheng, Su-xian Zhou, Li-li Huang, Xiao-ling Liu, Jing-qiong Xun, Yan Zhou

**Affiliations:** 1 Department of Endocrinology, The Affiliated Hospital of Guilin Medical University, Guilin, 541001 China; 2 The Graduate School of Guilin Medical University, Guilin, 541001 China; 3 Department of Respiratory Medicine, The Affiliated Hospital of Guilin Medical University, Guilin, 541001 China

**Keywords:** Hypertension in adults, Risk factor, Interaction

## Abstract

**Background:**

The prevalence of hypertension in adults is increasing each year and has become a main public health issue worldwide. We must consider the impact of both individual factors and interactions among these factors on hypertension in adults. This study was designed to elucidate the clinical and metabolic characteristics of the prevalence of hypertension in adults and to explore the risk factors and interactions among these factors in adults with hypertension.

**Methods:**

We used overall random sampling to conduct a cross-sectional survey of 6660 individuals undergoing a health check from July to November 2012, the subjects were aged 20 to 89 years, including 3480 men and 3180 women. The survey content included a questionnaire, anthropometry, laboratory measurements, and liver Doppler ultrasonography. The clinical and metabolic characteristics were compared between the cases (adult hypertensive patients) and the controls (normotensives). The classification tree model and the non-conditional logistic regression were used to analyze the interactions of risk factors for hypertension in adults.

**Results:**

In total, 1623 adult hypertensive patients (940 men and 683 women) were detected. The results showed that adult hypertensive patients were older and had higher levels of systolic blood pressure, diastolic blood pressure, body mass index, fasting plasma glucose, uric acid, triglycerides, total cholesterol, low-density lipoprotein cholesterol, and prevalence of non-alcoholic fatty liver disease (*P* < 0.001). The classification tree model comprising 5 layers, 39 nodes, and 20 terminal nodes showed that two variables, age and BMI, were closely related to hypertension in adults. The area under the receiver operating characteristic curve for classification tree model was 81.6 % (95 % *CI*: 80.6 % ~ 82.5 %). Both univariate and multivariate logistic regression analyses revealed that advanced age and high BMI had a significant positive interaction in terms of hypertension in adults. After controlling for confounding factors, the percentage of attributed interaction was 47.62 %.

**Conclusions:**

This study showed that age, BMI, UA, TG, and TC were closely associated with the risk of hypertension in adults, and the positive interaction effect between advanced age and high BMI was an important risk factor for the prevalence of hypertension in adults.

## Background

In recent years, with the rapid social and economic development and the change in people’s diets, the prevalence of hypertension has been increasing each year, especially in the adult population. Hypertension has become a chronic non-communicable disease that severely harms human health as well as affects the quality of life, and a main public health issue worldwide [1]. In 2000, approximately 972 million people had hypertension worldwide, and the prevalence of hypertension in adults was approximately 26.4 % (men: 26.6 %, women: 26.1 %). By 2025, the prevalence of hypertension in adults is expected to rise to 29.2 % (men: 29.0 %, women: 29.5 %) [2]. The prevalence of hypertension in adults varies across different regions [3] and is affected by genetic background and environmental factors. The incidence of hypertension was generally higher in developing countries than in developed countries [4], specifically in low- or middle-incomes countries, for example, the incidence of hypertension could reach up to 68.9 % in some African regions [5]. Hypertension is becoming one of the world’s most costly health conditions and poses an especially large burden of disease in some developing countries [6].

Cardiovascular disease (CVD) is a main cause of death in most developing countries [7], accounting for approximately 30 % of all deaths. As the most important risk factors for CVD, long-term hypertension could induce the endothelial damage of blood vessels, affect the blood circulation, and lead to a series of cardiovascular diseases, which is closely related to CVD morbidity and mortality [8]. In 2002, the World Health Organization estimated that 50 % of CVD cases may be attributed to high blood pressure. Additionally, hypertension is a main cause of stroke [9] and chronic kidney disease [10]. In China, which is a developing country with a large population, heart disease and cerebrovascular disease rank first and third as causes of death in people aged 40 years or above, and the primary risk factor for total mortality is hypertension [7].

The pathogenesis of hypertension in adults is complex, and the mechanism is unknown [11]. Identifying the factors that affect the prevalence of hypertension in adults and interactions among these factors have important implication for elucidating the clinical and metabolic pathogenesis and for taking preventive measures. Studies conducted among Chinese adults to investigate the prevalence of hypertension have majorly focused on genetics [12] and risk factors for hypertension. Our study was intended to investigate the clinical and metabolic factors affecting the prevalence of hypertension in adults, and to explore the main interactions among these factors for hypertension. Accordingly, in this study, we used the Classification and Regression Tree (CRT) to investigate the risk factors and interactions among these factors in adults with hypertension, and a non-conditional logistic regression model to quantitatively analyze the interactions, which were valuable for the primary care and disease prevention of hypertension in adults.

## Methods

### Study population

In this survey, we enrolled 7018 individuals who visited the Center of Health Examination, Affiliated Hospital of Guilin Medical University, Guilin, Guangxi, China, for health check from July to November 2012. Subjects were excluded from this study if they were younger than 18 years, had severe cardiovascular or cerebrovascular disease, were pregnant women, had cancer, or suffered liver or kidney impairment. All subjects signed the informed consent. On the day of the registration, each subject accepted a cross-sectional survey covered the questionnaire, anthropometry, laboratory measurements, and liver Doppler ultrasonography. A total of 6660 subjects with complete data were finally included in the study by the method of cluster random sampling, the study subjects were aged 20 to 89 years (mean: 48.07 ± 13.41 years), including 3480 men aged 48.37 ± 13.45 years and 3180 women aged 47.74 ± 13.35 years. Altogether 1623 adult hypertensive patients were detected and classified as the cases, and 5037 normotensives were classified as the controls. The data were statistically analyzed to investigate the risk factors and interactions among these factors in adults with hypertension. The study protocol was approved by the Ethics Committee of the Affiliated Hospital of Guilin Medical University (YXLL-2012-11).

### Data collection

Using the standardized questionnaire designed by medical specialists in Center of Health Examination, Affiliated Hospital of Guilin Medical University, to record the basic information in detail, including the basic situation of individuals (name, age, gender, nationality, etc.), the living habits (diet, smoking history, alcohol intake, etc.), the medical histories, the medications, the family histories of disease and physical activity.

### Anthropometry and laboratory measurements

Trained and qualified medical staffs used a SK-CK Ultrasonic Body Scale (Shenzhen, China) to measure height and weight. All subjects were asked to remove their hats and shoes and to stand erect for the height measurement (to the nearest 0.01 m); moreover, the subjects were asked to empty their bladders and to remove their shoes for the weight measurement (to the nearest 0.1 kg). The body mass index (BMI) was calculated as body weight (in kilograms) divided by height (in meters squared). All subjects rested for 5 to 10 min and then had their blood pressure measured in a sitting position. Before the measurement, all subjects were asked to avoid alcohol, smoking, tea, coffee, and strenuous exercise. The brachial artery blood pressure of the right arm was measured twice with a mercury sphygmomanometer (Yuwell; Danyang, China), 1 to 2 min apart. In cases with a significant between-measurement discrepancy, a third measurement was taken, and the mean value was used for the analysis (to the nearest 1 mmHg).

A venous blood samples (5 mL) of the subjects were collected after at least 10 h of fasting to measure blood biochemical indicators: uric acid (UA) was measured by the enzymatic assay, triglycerides (TG) was measured by the a glycerol phosphate oxidase-peroxidase assay, total cholesterol (TC) was measured by the cholesterol oxidase assay, low-density lipoprotein cholesterol (LDL-C) was measured by the surfactant assay, high-density lipoprotein cholesterol (HDL-C) was measured by the phosphotungstic acid-magnesium assay, and fasting plasma glucose (FPG) was measured by the glucose oxidase assay. Another blood sample was drawn at 120 min after an oral glucose load (75 g) to measure 2-h plasma glucose (2hPG) in the oral glucose tolerance test (OGTT). All blood samples were measured within 2 h of collection using the Roche Cobas C501 (Basel, Switzerland) automatic biochemical analyzer. Moreover, the specialist physicians used Mindray DC-6 Expert type II ultrasound system (Shenzhen, China) to examine the liver.

### Quality control

The trained staffs performed a questionnaire survey and recorded the basic information in detail; the blood pressure, weight and height were measured by the sphygmomanometer and scale, which were centrally calibrated; the EpiData software, version 3.0.2 (Odense, Denmark), was used to record the data and perform logistic testing. The laboratory analysis center of Affiliated Hospital of Guilin Medical University was responsible for whole laboratory testing and quality control.

### Diagnosis and classification

Hypertension was diagnosed in accordance with Chinese and international guidelines, as follows: systolic blood pressure ≥ 140 mmHg and/or diastolic blood pressure ≥ 90 mmHg, or a previous diagnosis of hypertension (even if the blood pressure was controlled at the normal level with antihypertensive drugs) [13]; The diagnosis of the hyperuricemia was UA > 420 μmol/L and UA > 360 μmol/L in male and in female, respectively; Diabetes was defined based on the WHO criteria (1999) [14]: FPG ≥ 7.0 mmol/L and/or 2hPG ≥ 11.1 mmol/L in OGTT. BMI was classified based on the result of classification tree. Dyslipidemia was considered if TC ≥ 5.18 mmol/L and/or TG ≥ 1.70 mmol/L and/or LDL-C ≥ 3.37 mmol/L and/or HDL-C < 1.04 mmol/L [15]. The diagnostic criteria for non-alcoholic fatty liver disease (NAFLD) was as follows: no history of heavy drinking; no other NAFLD-inducing diseases such as drug-induced liver disease or viral hepatitis, and liver ultrasound imaging findings suggestive of a diffuse fatty liver [16].

### Statistical analysis

All analyses were performed with SPSS software (version 18.0, SPSS, Chicago, IL, USA), and MedCalc software (version 11.4.2.0, Mariakierke, Belgium). Continuous variables with normal distributions were expressed as mean ± standard deviation, and analyzed with the *t*-test. Categorical variables were expressed as percentages and analyzed with the Chi-square (*χ*^*2*^)-test. The age structure from the 2010 Sixth China Population Census was used as the standard, and the direct standardized method was used to adjust the overall prevalence of hypertension in adults. All tests for statistical significance were two-tailed, and *P*-value < 0.05 was considered statistically significant.

The CRT model is a nonparametric analysis which is applied to analyze potential interactions between continuous or categorical variables. The minimum sample size was 100 for the parent nodes of the classification tree and 50 for the child nodes. CRT analysis identified optimal cut-off points for continuous discriminating variables in the sets of growth limits and significance levels. The splitting nodes and merging criterions were 0.05 at each stage. The tree branched and grew iteratively until a termination criterion was met or no further significant improvement in the classification of study subjects was possible. CRT selected outcome variables with close associations with adult hypertensive patients from all influencing factors, and visually displayed their interactions in the form of a tree diagram. The accuracy performance of the classification tree model was evaluated by the receiver operating characteristic (ROC) curve. ROC curve was obtained according to the predicted probability of CRT model for hypertension, the optimal cut-off value determined by the maximal Youden index (sensitivity + specificity - 1) was identified to calculate the sensitivity and specificity with accurate 95 % confidence intervals (CI). The area under the curve (AUC) derived from ROC analysis could reflect the accuracy capacities of CRT model, which ranges from 0.5 to 1.0. The AUC value greater than 0.5 and less than or equal to 0.7 indicates a lower accuracy capacity, the AUC value greater than 0.7 and less than or equal to 0.9 means a medium accuracy capacity, the AUC value greater than 0.9 reveals a higher accuracy capacity.

Non-conditional logistic regression model is a traditional analysis method, and usually appropriated for investigating the relationships between the dependent variable and independent variables, and identifying risk or protective factors for the dependent variable, which is used for binary variables. In our study, the non-conditional logistic regression model was performed to analyze the interactions among influencing factors. The cut-off points of the classification tree were used to assign age and BMI values, and the values of the remaining factors were assigned on the basis of professional expertise. Significant factors shown in the univariate logistic regression model were used for multivariate logistic regression analysis with the stepwise forward method. In the quantitative analysis of the interactions, OR (A^0^B^0^) indicated the odds ratio (OR) in the absence of both advanced age and high BMI, OR (AB^0^) indicated the OR in the presence of advanced age only, OR (A^0^B) indicated the OR in the presence of high BMI only, and OR (AB) indicated the OR in the presence of both advanced age and high BMI. Attributed interaction [I (AB)] = OR (AB) - OR (AB^0^) - OR (A^0^B) + OR (A^0^B^0^), the percentage of attributed interaction (AP) = I (AB)/OR (AB), the percentage of attributed interaction between pure factors [AP* (AB)] = I (AB)/[OR (AB) - OR (A^0^B^0^)], and synergy index (S) = [OR (AB) - 1]/[OR (AB^0^) – 1 + OR (A^0^B) - 1].

## Results

### Comparison of the clinical and metabolic characteristics

In total, 1623 adult hypertensive patients of which 31.1 % had NAFLD were detected and classified as the cases, aged 57.70 ± 11.32 years, including 940 men (57.9 %) and 683 women (42.1 %). The crude prevalence of hypertension in adults was 24.4 % (men: 27.0 %; women: 21.5 %). The population-standardized prevalence in China was 20.5 % (standardized prevalence: men: 22.3 %; women: 18.5 %). The remaining 5037 normotensives of which 15.3 % had NAFLD were classified as the controls, age 44.97 ± 12.53 years, including 2540 men (50.4 %) and 2497 women (49.6 %). Significant differences in gender (*χ*^*2*^ = 27.605, *P* < 0.001) and NAFLD incidence (*χ*^*2*^ = 197.863, *P* < 0.001) were observed between the two groups. As shown in Table 1, adult hypertensive patients were older and had higher levels of SBP, DBP, BMI, FPG, UA, TG, TC, and LDL-C (*P* < 0.001), whereas they had a significantly lower level of HDL-C (*P* < 0.001).

**Table 1:** The clinical and metabolic characteristics of two groups

Variables	Cases	Controls	*t* /*χ* ^*2*^ value	*P*-value^a^
N	1623	5037	-	-
Age (years)	57.70 ± 11.32	44.97 ± 12.53	36.410	0.000
Sex (male/female)	940/683	2540/2497	27.605	0.000
NAFLD (%)	31.10	15.30	197.863	0.000
SBP (mmHg)	150.17 ± 13.83	118.77 ± 12.18	87.299	0.000
DBP (mmHg)	91.45 ± 9.47	73.86 ± 8.78	68.837	0.000
BMI (kg/m^2^)	25.52 ± 2.96	23.71 ± 3.09	20.773	0.000
FPG (mmol/L)	5.84 ± 1.22	5.38 ± 0.91	16.332	0.000
2hPG (mmol/L)	7.15 ± 1.40	7.08 ± 1.96	1.531	0.126
UA (μmol/L)	351.31 ± 87.18	318.60 ± 84.52	13.456	0.000
TG (mmol/L)	1.93 ± 1.38	1.47 ± 1.10	13.613	0.000
TC (mmol/L)	5.18 ± 0.89	4.86 ± 0.87	12.954	0.000
LDL-C (mmol/L)	3.47 ± 0.85	3.11 ± 0.85	14.793	0.000
HDL-C (mmol/L)	1.38 ± 0.37	1.46 ± 0.40	−7.078	0.000

Variables are shown as mean ± standard deviation (SD), number (n) or percentage (%). The cases were referring to the adult hypertensive patients, and the controls were referring to the normotensives

*Abbreviations*: *N* number, *NAFLD* non-alcoholic fatty liver disease, *SBP* systolic blood pressure, *DBP* diastolic blood pressure, *BMI* body mass index, *FPG* fasting plasma glucose, *2hPG* 2-h plasma glucose, *UA* uric acid, *TG* triglyceride, *TC* total cholesterol, *LDL-C* low-density lipoprotein cholesterol, *HDL-C* high-density lipoprotein cholesterol

^a^ *P*-values compare clinical and metabolic characteristics between 1623 cases and 5037 controls, using either Independent-Samples *t*-test or *χ* ^*2*^-test

### Classification tree model analysis

**Fig. 1:**
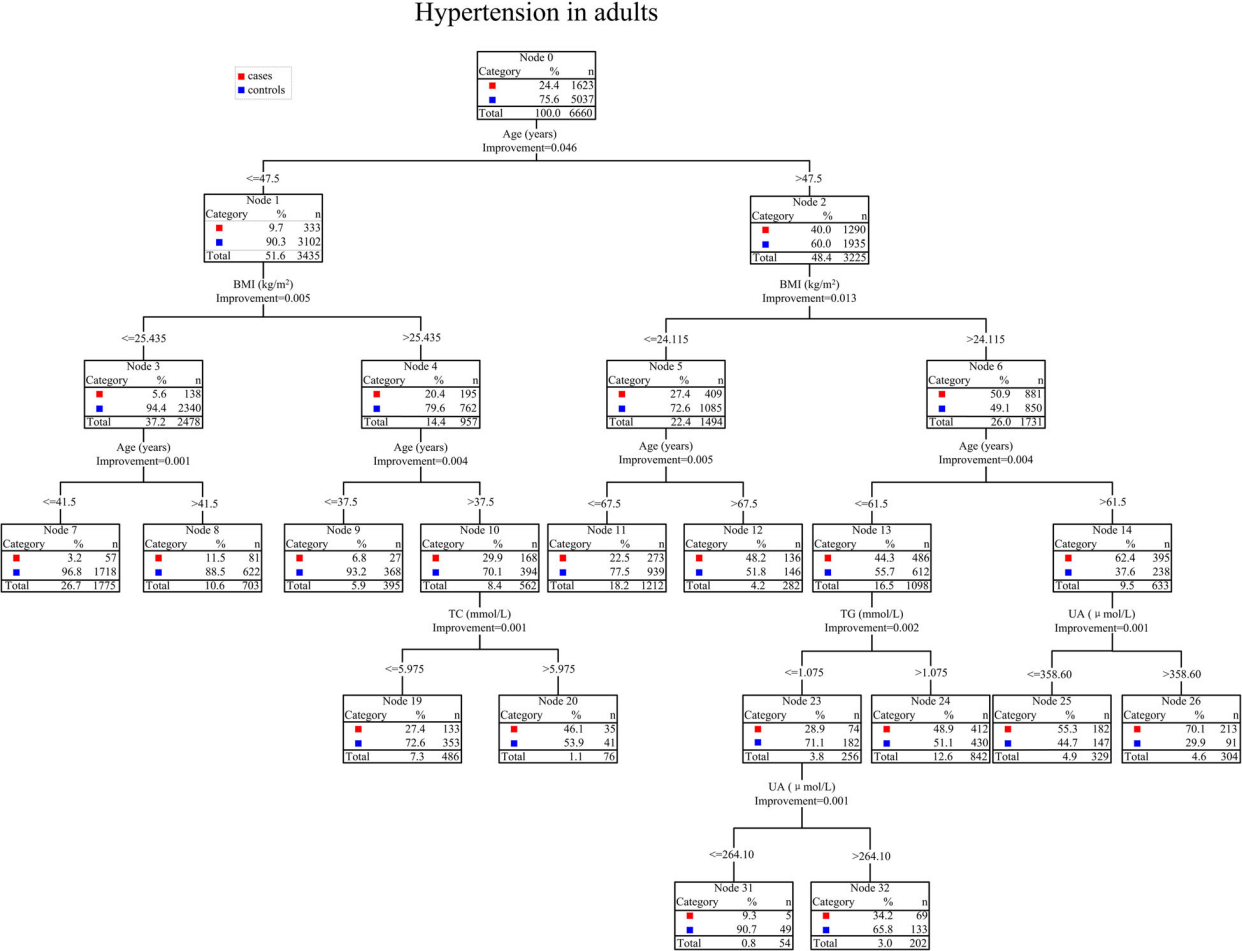
The classification tree model for hypertension in adults. Data are shown in the percentage (%) and number (n). Continuous variables were divided into categorical variables after the classification tree was grown and pruned based on the restriction of the minimum sample size of the root (100) and child (50) nodes. The figure showed only variables with close associations with hypertension in adults. Abbreviations: n, number; BMI, body mass index; UA, uric acid; TG, triglyceride; TC, total cholesterol

Classification tree model was used to analyze factors affecting the prevalence of hypertension in adults, the cases were assigned a value of “1” and the controls were assigned a value of “0” in the model. After growth and trimming based on the limitation of the minimum sample size of the root and child nodes, the classification tree model comprised 5 layers, 39 nodes, and 20 terminal nodes as well as revealed only five variables with a significant impact on hypertension in adults—age, BMI, UA, TG, and TC—with interactions among variables (Fig. 1). In particular, the interaction between age and BMI was closely related to the prevalence of hypertension in adults (Fig. 1). We had trimmed the figure due to the original layout was bigger, as shown in Fig. 1.

**Fig. 2:**
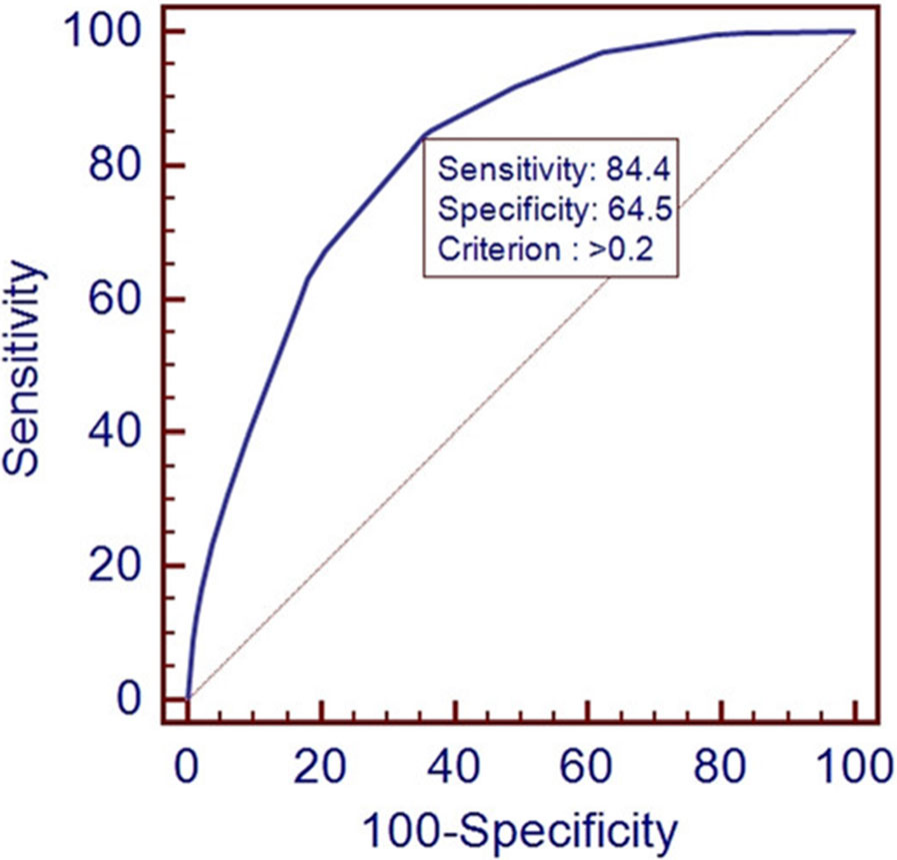
The receiver operating characteristic (ROC) curve of CRT model for hypertension. The area under the ROC curve (AUC) value was 81.6 %, the optimal cut-off point of 0.2 marked with a sensitivity of 84.4 % and a specificity of 64.5 %

We used the predicted probability derived from CRT analysis for hypertension as the test variable to establish a ROC curve, and the value of state variable was hypertension, the ROC curve for CRT yielded the optimal cut-off value (0.2) based on the maximal Youden index (48.95 %), and the AUC value was 81.6 % (95 % *CI*: 80.6 % ~ 82.5 %, *P* < 0.01) with a sensitivity of 84.41 % (95 % *CI*: 82.6 % ~ 86.1 %) and a specificity of 64.54 % (95 % *CI*: 63.2 % ~ 65.9 %) for hypertension, as shown in Fig. 2 and Table 2.

**Table 2:** Cut-off criterion, sensitivity, specificity, AUC and Youden index of ROC curve for CRT model

Parameters	ROC curve of CRT model
Cut-off criterion	0.2
Sensitivity % (95 % *CI*)	84.41 (82.6 ~ 86.1)
Specificity % (95 % *CI*)	64.54 (63.2 ~ 65.9)
AUC % (95 % *CI*)	81.6 (80.6 ~ 82.5)^*^
Youden index %	48.95

Sensitivity, specificity, AUC and Youden index of ROC curve are shown as percentages (%), and numbers in parentheses are 95 % CI

*Abbreviations*: *ROC* receiver operating characteristic, *CRT* classification and regression tree, *CI* confidence interval, *AUC* area under the curve

^*^ *P* < 0.01

### The interaction effects of risk factors for hypertension in adults

The binary variable of adult hypertension was defined as the dependent variable, and dichotomous variables of age, BMI, UA, TG, and TC were defined as independent variables. Age was most closely related to hypertension in adults and was divided into dichotomous variables according to the cut-off points (47.5 years) of the classification tree. The incidence of hypertension in adults was higher in subjects aged > 47.5 years, and thus the use of 24.115 kg/m^2^ as the cut-off point of BMI level, which was consistent with the classification criteria of Chinese BMI guidelines [17]. The remaining 3 influencing factors were assigned a value according to professional guidelines (Table 3). Age and BMI were two factors most closely related to the prevalence of hypertension in adults. To further investigate the interaction between age and BMI on hypertension in adults, non-conditional logistic regression was used for quantitative analysis of the interaction between age and BMI. Univariate logistic regression showed that the risk of hypertension in adults was 21.248 times higher in people with advanced age and high BMI than in those without advanced age and high BMI. In addition, the risk of hypertension in adults was 1.895 times higher in people with high UA, 2.083 times higher in those with high TG, and 2.040 times higher in those with high TC (Table 4).

**Table 3:** Variables and assignments in non-conditional logistic regression model

Variables	Assignments
Hypertension	No = 0; Yes = 1
Age (years)	≤47.5 = 0; > 47.5 = 1
BMI (kg/m^2^)	≤24.115 = 0; > 24.115 = 1
UA (μmol/L)	Male ≤ 420, Female ≤ 360 = 0; Male > 420, Female > 360 = 1
TG (mmol/L)	<1.70 = 0; ≥ 1.70 = 1
TC (mmol/L)	<5.18 = 0; ≥ 5.18 = 1

*Abbreviations*: *BMI* body mass index, *UA* uric acid, *TG* triglyceride, *TC* total cholesterol

**Table 4:** Non-conditional logistic regression analysis for hypertension in adults

Variables^a^		Controls (%)	Cases (%)	*OR* (95 % *CI*)	
		*n* = 5037	*n* = 1623	Unadjusted	Adjusted^b^
BMI: (−)	age: (−)	1927 (38.3)	94 (5.8)	1.000 (reference)	1.000 (reference)
(−)	(+)	1085 (21.5)	409 (25.2)	7.728 (6.102 ~ 9.787)^*^	6.891 (5.429 ~ 8.748)^*^
(+)	(−)	1175 (23.3)	239 (14.7)	4.170 (3.249 ~ 5.351)^*^	3.301 (2.556 ~ 4.264)^*^
(+)	(+)	850 (16.9)	881 (54.3)	21.248 (16.925 ~ 26.675)^*^	17.548 (13.931 ~ 22.103)^*^
Hyperuricemia: no		4453 (88.4)	1300 (80.1)	1.000 (reference)	1.000 (reference)
Yes		584 (11.6)	323 (19.9)	1.895 (1.632 ~ 2.200)^*^	1.478 (1.246 ~ 1.752)^*^
High TG: no		3717 (73.8)	933 (57.5)	1.000 (reference)	1.000 (reference)
Yes		1320 (26.2)	690 (42.5)	2.083 (1.853 ~ 2.340)^*^	1.365 (1.191 ~ 1.564)^*^
High TC: no		3414 (67.8)	824 (50.8)	1.000 (reference)	1.000 (reference)
Yes		1623 (32.2)	799 (49.2)	2.040 (1.820 ~ 2.286)^*^	1.411 (1.241 ~ 1.604)^*^

*Abbreviations*: *BMI* body mass index, *TG* triglyceride, *TC* total cholesterol, *OR* odds ratio, *CI* confidence interval

^a^Cut-offs for high BMI = 24.115 kg/m^2^, advanced age = 47.5 years, hyperuricemia = 420 μmol/L and 360 μmol/L in male and in female respectively, high TC = 5.18 mmol/L and high TG =1.7 mmol/L. Those with normal values were considered as the reference group

^b^Multivariate logistic regression analysis adjusted for all other variables in the Table 4

^*^ *P* < 0.001 compared with the reference group

After correction of the five factors related to the prevalence of hypertension in adults (α_in_ = 0.10, α_out_ = 0.15), the results of multivariate logistic regression analysis showed that age, BMI, age × BMI, high TC, high TG, and hyperuricemia were risk factors for the prevalence of hypertension in adults (*P* < 0.01). Moreover, the risk of hypertension in adults was 17.548 times higher in people with advanced age and high BMI than in those without advanced age and high BMI. In addition, the risk of hypertension in adults was 1.478 times higher in people with high UA, 1.365 times higher in those with high TG and 1.411 times higher in those with high TC (Table 4).

For further quantitative analysis of the interaction, relevant parameters [I (AB), AP, AP*(AB), and S] were calculated based on the results of univariate and multivariate logistic regression analyses, as shown in Table 5.

**Table 5:** Interactions between advanced age and high BMI

	Univariate logistic regression	Multivariate logistic regression^a^
I (AB)	10.35	8.36
AP	48.71 %	47.62 %
AP*(AB)	51.12 %	50.50 %
S	2.05	2.02

*Abbreviations*: *I (AB)* attributed interaction, *AP* percentage of attributed interaction, AP*(AB) percentage of attributed interaction between pure factors, *S* synergy index

^a^The multivariate logistic regression model adjusted for age, BMI, UA, TG, and TC

## Discussion

National Nutrition and Health Survey showed the prevalence of hypertension in China aged 18 years or above was 18.8 % [18], the overall morbidities of northern and southern China was investigated to be 25.8 and 20.4 % respectively [19]. Our study revealed the crude and population-standardized morbidity of hypertension in Guilin adults was 24.4 and 20.5 % respectively, which was lower than most of the northeastern cities of China [20], However, compared with morbidities in other cities of the same southwestern areas (Guangzhou: 11.8 %; Nandan county of Guangxi: 16.45 %) [21, 22], the prevalence of hypertension in adults was higher in Guilin. The study compared clinical and metabolic characteristics of hypertensive and normotensive adults in Guilin. Hypertensive patients were older and had higher levels of BMI, FPG, UA, TG, TC, LDL-C, and NAFLD morbidity, whereas they had a significantly lower level of HDL-C, indicating that adults with hypertension might easily suffer from metabolic disorders such as overweight, dyslipidemia, hyperuricemia, hyperglycemia, NAFLD, et al.

Therefore, a further study by the classification tree analysis and non-conditioned logistic regression model on the meaningful factors was conducted to make clear the influence on the hypertension in adults. The classification tree model is primarily used to analyze discrete dependent variables, whereas the regression tree is primarily used to predict continuous dependent variables and to select optimal branch thresholds by growing and pruning the tree [23]. Our study used the classification tree to survey the factors affecting the prevalence of hypertension in adults and the interactions among these factors. The classification tree model visually shows the results in the form of a tree diagram, processes the interactions among variables well, indicates the characteristics of populations prone to disease, analyzes specific populations affected by the variable, and intervenes high-risk populations. Compared with the traditional analysis method, the classification tree model is not subject to the potential co-linear effect of variables during the analysis [24]. We used ROC curve to evaluate the accuracy of CRT analysis, the cut-off value, sensitivity, specificity, and AUC of ROC curve was 0.2, 84.41, 64.54, and 81.6 %, respectively, indicating that the CRT model had a medium accuracy performance.

The first layer of the classification tree was age. Previous investigations have shown that advanced age is a risk factor for hypertension and the prevalence of hypertension in adults increases with age [25, 26]. Our study also indicated the age was the most important factor affecting hypertension in adults, and the cut-off point of age was 47.5 years. The results showed that the incidence of hypertension in adult was significantly higher in subjects aged > 47.5 years (40.0 %) compared to subjects aged ≤ 47.5 years (9.7 %). Studies have also shown that the prevalence of hypertension is higher in people aged 40 or above [27], which is consistent with our investigation. The prevalence of hypertension was higher in middle-aged and elderly populations because of the declining functions of the body with age, such as decreased arterial elasticity, luminal narrowing, and lipid and calcium deposits in vessel walls [28]—all of these factors may lead to hypertension in adults. Accordingly, the middle-aged and elderly populations are key populations for hypertension prevention and control, and we should promote health education and take preventive measures to reduce the prevalence of hypertension in adults.

The second layer of the classification tree was the BMI (cut-off point: 24.115 kg/m^2^), which was consistent with the Chinese criteria for being overweight or obese [17]. Hence, the results indicated that the prevalence of hypertension in adults was significantly higher in people who were overweight or obese (BMI > 24.115 kg/m^2^), which was consistent with other Chinese and international researches [18, 29, 30]. Because of the change in people’s diet and decrease in exercise intensity, more and more individuals are becoming overweight or obese, which is closely related to CVD [31]. A study [32] investigating the risk factors for microvascular and macrovascular diseases revealed that patients with the history of obesity were more likely to exhibit arterial intimal thickening, probably because free fatty acids secreted by fat cells induce the production of a large amount of inflammatory cytokines [33], resulting in vascular injury, atherosclerosis, and macrovascular disease. In addition, obesity could cause leptin resistance and increase activity of the renin-angiotensin-aldosterone system, which ultimately cause increased peripheral vascular resistance and elevated blood pressure. Therefore, we should promote a healthy diet with regular physical exercise to control the incidence of overweight and obesity, which will have positive effects on hypertension in adults prevention and treatment.

Age was also the factor at the third layer of the classification tree model, suggesting that age was closely related to hypertension in adults, and the strong interaction between age and BMI had an impact on the prevalence of hypertension in adults. So we used the logistic regression model to quantitatively analyze the interaction to compensate for the inadequacy of the classification tree. Both univariate and multivariate logistic regression models suggested that advanced age and high BMI were independent risk factors for hypertension in adults, and advanced age and high BMI had a positive interaction with respect to the prevalence of hypertension in adults [OR (AB) > OR (AB^0^) + OR (A^0^B) - OR (A^0^B^0^)], which was consistent with the classification tree model. Next, we used the additive model (more meaningful from the perspective of public health) [34] to investigate the interaction effect between advanced age and high BMI on hypertension in adults. The univariate logistic regression model showed that the percentage of attributed interaction was 48.71 %, suggesting that 48.71 % of the hypertension risk in adults was due to the interaction between advanced age and high BMI. After controlling for confounding factors, the percentage of attributed interaction was 47.62 %, suggesting that 47.62 % of the hypertension risk in adults was due to the interaction between advanced age and high BMI. Both results showed that advanced age and high BMI had a significant synergistic effect on the prevalence of hypertension in adults, and the risk of hypertension in adults was significantly higher if both risk factors were present relative to if only one factor was present.

Furthermore, the fourth and fifth layers of the classification tree suggested that the interactions between UA, TG, and TC also had an impact on hypertension in adults. Both univariate and multivariate logistic regression analyses showed that high TC, high TG, and high UA were independent risk factors for hypertension in adults. High TG and/or high TC are manifestations of dyslipidemia [16], in which excess lipids accumulate in the vascular endothelium, which promotes thrombosis and leads to atherosclerosis and hypertension. Dyslipidemia is also an important risk factor for heart disease [35], arteriosclerosis, and chronic kidney disease. Studies have shown that serum UA is an important factor for hypertension [36]. Hyperuricemia inhibits endothelial nitric oxide synthesis and release, and stimulates the renin-angiotensin-aldosterone system; moreover, urate crystals may damage the arterial intima, leading to oxidative stress, vascular inflammation, and elevated blood pressure [37]. Lowering serum UA helps control the cardiovascular risk factors for hypertension, chronic kidney disease [38], abnormal blood lipid levels [39], etc. Therefore, we must control serum UA and lipid levels, especially in patients with gout and hyperlipidemia, to proactively prevent and improve hypertension in adults.

### Limitations

However, our present study also had some limitations. First, all included adult subjects were from Guilin, China, and thus they may not represent the whole Chinese adults. Second, further prospective studies are needed to survey the causal relationship between the risk factors and hypertension in adults. Third, this was an epidemiological study, so we cannot clarify the exact mechanism underlying the between-factor interaction.

## Conclusions

This study showed that age, BMI, UA, TG, and TC were closely associated with the risk of hypertension in adults. Advanced age and high BMI were important risk factors for hypertension and had a positive interaction effect for the prevalence of hypertension in adults. The study has important implications for understanding the pathogenesis of hypertension, taking appropriate preventive measures, and conducting additional studies to clarify the relevant mechanisms of action. Incidence of hypertension in adults can be prevented or reduced by targeting the risk factors associated with hypertension eg BMI, UA, TG, and TC.

## Abbreviations

2hPG: 2-h plasma glucose
AP*(AB): Percentage of attributed interaction between pure factors
AP: Percentage of attributed interaction
AUC: Area under the curve
BMI: Body mass index
CI: Confidence interval
CRT: Classification and regression tree
CVD: Cardiovascular disease
DBP: Diastolic blood pressure
FPG: Fasting plasma glucose
HDL-C: High-density lipoprotein cholesterol
I (AB): Attributed interaction
LDL-C: Low-density lipoprotein cholesterol
N: Number
NAFLD: Non-alcoholic fatty liver disease
OGTT: Oral glucose tolerance test
OR: Odds ratio
ROC: Receiver operating characteristic
S: Synergy index
SBP: Systolic blood pressure
TC: Total cholesterol
TG: Triglycerides
UA: Uric acid
